# Fractioning and Compared ^1^H NMR and GC-MS Analyses of Lanolin Acid Components

**DOI:** 10.3390/molecules28041635

**Published:** 2023-02-08

**Authors:** Camillo Morano, Michele Dei Cas, Roberta F. Bergamaschi, Erika Palmisano, Marco Pallavicini, Cristiano Bolchi, Gabriella Roda, Sara Casati

**Affiliations:** 1 Department of Pharmaceutical Sciences, Università degli Studi di Milano, 20133 Milan, Italy; 2 Department of Health Sciences, Università degli Studi di Milano, 20142 Milan, Italy; 3 Department of Biomedical, Surgical and Dental Sciences, Università degli Studi di Milano, 20133 Milan, Italy

**Keywords:** fatty acids, α-hydroxy fatty acids, biomasses, lanolin, GC-MS, NMR spectroscopy

## Abstract

The management of food and food-related wastes represents a growing global issue, as they are hard to recycle and dispose of. Foremost, waste can serve as an important source of biomasses. Particularly, fat-enriched biomasses are receiving more and more attention for their role in the manufacturing of biofuels. Nonetheless, many biomasses have been set aside over the years. Wool wax, also known as lanolin, has a huge potential for becoming a source of typical and atypical fatty acids. The main aim of this work was to evaluate and assess a protocol for the fractioning of fatty acids from lanolin, a natural by-product of the shearing of sheep, alongside the design of a new and rapid quantitative GC-MS method for the derivatization of free fatty acids in fat mixtures, using MethElute™. As the acid portion of lanolin is characterized by the presence of both aliphatic and hydroxylated fatty acids, we also evaluated a procedure for the parting of these two species, by using NMR spectroscopy, benefitting of the different solubilities of the components in organic solvents. At last, we evaluated and quantified the fatty acids and the α-hydroxy fatty acids present in each attained portion, employing both analytical and synthetic standards. The performed analyses, both qualitative and quantitative, showed a good performance in the parting of the different acid components, and GC-MS allowed to speculate that the majority of α-hydroxylated fatty acids is formed of linear saturated carbon chains, while the totality of properly said fatty acids has a much more complex profile.

## 1. Introduction

The waste associated to the food supply chain is receiving more and more general interest, to the end of renewing the production lane with a particular eye to the reduction of pollution and squandering. In 2021, the United Nations released the UNEP Food Waste Index Report, whereas it was reported that 931 million tonnes of food were wasted in the previous year, including the discarded food from households, retail establishments, and the food service industry [1]. It is evident that the amount of animal parts that remain completely unused is incredibly high. These scraps are usually referred to as biomasses and they are often used in industry, with roles that vary from energy production, in the forms of biofuels and biopower sources, to the synthesis of new chemical entities or the upgrade of already existent synthetic ways (bioproducts) [2,3,4]. Biomasses are not considered food per se, but many of them derive from food-related macro-organisms.

Fatty acids can constitute a major part of food wastes and inedible parts associated to food production; in fact, these biomasses can reach up to 70% of the total lipid content [5]. A few examples of such scraps are represented by waste cooking oils, peels and seeds of vegetables, and animal fats. The grease of sheared fleeces, also known as wool wax or lanolin, is a wax secreted by the sebaceous glands of sheep and is a fitting example of an inevitable by-product which, in EU only, can reach 30 thousand tons yearly [6]; in fact, right after shearing, wool needs to be thoroughly washed to remove any impurity that could result in an adulteration of the final wool used in the textile industry, with a process called scouring. Alongside these impurities, made up of dirt, dead skin, and vegetable matter, freshly sheared wool is particularly enriched of wool wax [7]. The disposal of excess lanolin, on the other hand, is very problematic, as the burning of this biomass is an unsustainable practice; foremost, greasy wool extracts could represent a renewable waste biomass, unique in properly said fatty acids (FAs) and, above all, α-hydroxy fatty acids (α-HFAs) richness. According to the literature, the main component of lanolin is a mixture of esters between long chain saturated alcohols and carboxylic acids (up to 97%), whose main classes are FAs, α-HFAs, and a small fraction of ω-HFAs. Secondary components are free fatty alcohols, free FAs (up to 8%), and sterols [8,9,10,11,12].

FAs are often indirectly analysed in gas chromatography coupled to mass spectrometry (GC-MS) because of their tendency to polymerization, cracking, and deacidification at high temperatures, though recent insights also rely on their analysis in LC-MS/MS [13,14]. The most common derivatization method for GC-MS resides in the conversion of FAs to their respective Fatty Acid Methyl Esters (FAMEs), using different agents, both alkaline and acid. Alkaline derivatization techniques allow a rapid derivatization of FAs, though they are not suitable for the derivatization of free FAs, but only for those involved in the formation of more complex lipids (e.g., triacylglycerols). On the other hand, acid derivatization methods mostly rely on the use of methanolic hydrochloric acid, acetyl chloride, sulfuric acid, and boron trifluoride, but these solutions require longer derivatization times and high temperatures; nonetheless, acid derivatizations often require the extraction of the analytes using very nonpolar solvents (i.e., hexane). Foremost, these reactions and procedures can be dangerous for the operator [15,16,17].

The aim of this work was to build a procedure for the recovery of carboxylic acids from wool wax and their fractioning into FAs and α-HFAs. Within this context, proton nuclear magnetic resonance (^1^H NMR) spectroscopy analysis proved to be a useful tool for a quick and as accurate as possible qualitative and semi-quantitative analysis of the composition of the different portions. Foremost, we developed two new GC-MS methods for the identification and quantitation of both FAs and α-HFAs, using different derivatizing agents, which allowed a rapid and safe conversion of the analytes.

## 2. Results

### 2.1. Method Validation

#### 2.1.1. Calibration Range and Linearity

Standard calibration curves (n = 3) were obtained by spiking standard solutions, as described in paragraph 3.2. The calibration ranges were set as listed in Table 1. All calibration curves showed good linearity (r^2^ >0.994) for all the analysed compounds over the entire investigated range when using linear correlation. The mean percentage coefficient of variations (%CV), limits of detection (LOD), and limits of quantitation (LOQ) have been calculated for all analytes and are listed in Table 2.

#### 2.1.2. Accuracy and Precision

Regarding precision and accuracy, the method showed satisfactory performance in terms of both repeatability and reproducibility, presenting %CV values below 15%. The same results were obtained for accuracy studies. Both precision and accuracy for all the analytes were within acceptable limits, as shown in Table 3.

### 2.2. Lanolin Acid Components Fractioning

**Figure 1:**
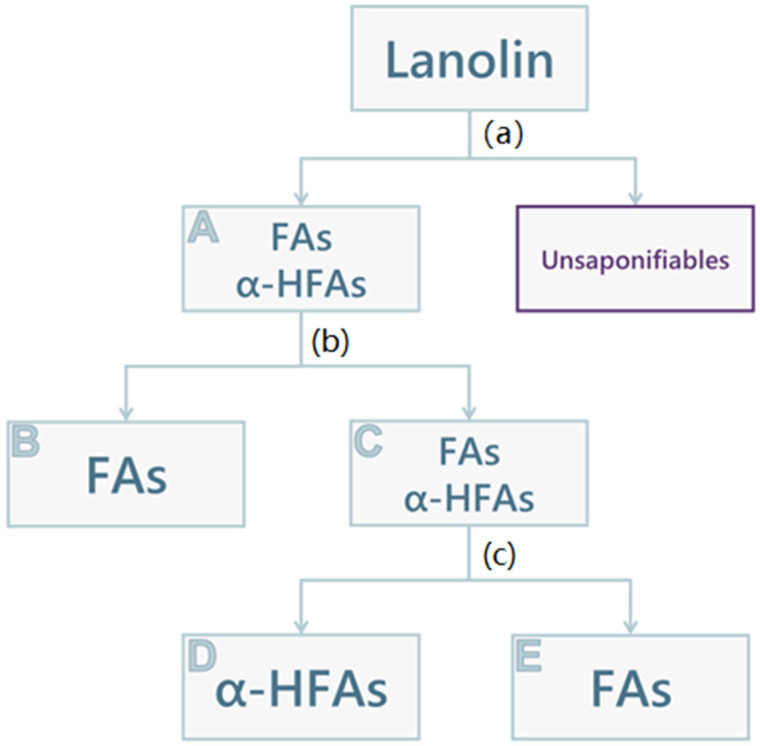
Schematic representation of lanolin acid components fractioning. (**a**) KOH 10% (*w*/*v*), reflux, 48 h; washing with ethyl acetate, acidification with HCl 30%, filtration, and recovery of the precipitate (**A**); (**b**) Methanol, rt, 2 h; filtration, recovery of the solid (**B**), and drying under vacuum of solvent to give solid (**C**); (**c**) Cyclohexane, rt, o.n; filtration, recovery of the solid (**D**), and drying under vacuum of solvent to give solid (**E**).

The qualitative and semi-quantitative compositions of the extracts were evaluated using ^1^H NMR spectroscopy analysis. FAs and α-HFAs can be distinguished by two singular peaks, as previously reported [18]: FAs are characterised by a triplet at 2.35 ppm (-CH_2_-CO-, 2H, Figure S1), α-HFAs by a doublet of doublets at 4.26 ppm (-CHOH-CO-, 1H, Figure S2). The extraction of acid components of lanolin was performed in triplicate, as follows, and is schematically pictured in Figure 1. Lanolin components were saponified using a heated aqueous solution of KOH 10% (*w/v*). After cooling the mixture, all its unsaponifiable components were extracted using ethyl acetate and then discarded. The alkaline phase was acidified for the precipitation of the aforementioned acid components. The attained solid (A, 3.5 g) consisted of a mixture of FAs and α-HFAs, with an α-HFAs molar fraction of about 0.43 (Table 4). At this point, the separation of FAs and α-HFAs was performed by using the different solubilities of the components of interest in different organic green solvents, namely methanol and cyclohexane. Therefore, the solid (A) was treated with methanol to attain a precipitate that, according to ^1^H NMR spectroscopy, is exclusively composed of FAs (B, 1.2 g). The product that remained in the methanolic solution was recovered by solvent drying, forming a solid wax (C, 2 g), that was again mainly composed of a mixture of α-HFAs and FAs, this time with a slightly higher α-HFAs molar fraction (0.52, Table 4). This last portion was then treated with cyclohexane: another precipitate was formed (D, 0.75 g), constituted of α-HFAs only, as appreciable in Table 4. The remaining FAs (E, 1 g) were recovered once again by evaporation of solvent under reduced pressure; however, the ^1^H NMR spectrum still showed a small fraction of α-HFAs (molar fraction: 0.28, Table 4). The spectra of the different portions (A–E) can be found in the supporting information section (Figures S3–S7). 

### 2.3. GC-MS Analysis of Lanolin Acid Components in Portions A–E

**Figure 2:**
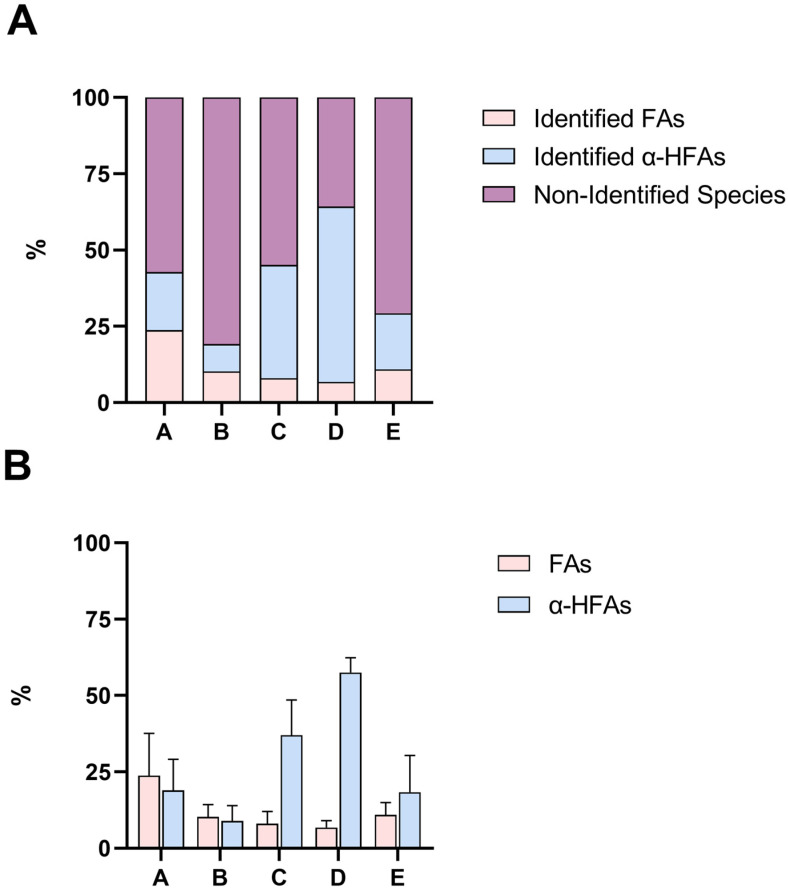
Panel (**A**) Percentages (*w*/*w*) of identified FAs (pink), identified α-HFAs (blue), and non-identified (purple) species. Results are graphed as mean. Panel (**B**) Percentages (*w*/*w*) of FAs (pink) and α-HFAs (blue) in the analysed samples, according to GC-MS. Results are graphed as mean and SD. In both panels, A–E refer to the different portions of lanolin, whereas A is composed of a mixture of FAs and α-HFAs, B of almost solely FAs, C of a mixture of FAs and α-HFAs, D of solely α-HFAs, and E of mainly FAs, as appraisable by ^1^H NMR spectroscopy (Figures S3–S7). See Figure 1 for reference.

The 15 different portions attained from the fractioning of the lanolin acid components (A-E) were analysed for the identification and quantitation of both FAs and α-HFAs using the hereby proposed GC-MS methods. The quantitation of FAs was achieved using n. 7 analytical standards of even saturated chain FAs, ranging from lauric acid (FA 12:0) to lignoceric acid (FA 24:0), and n. 4 analytical standards of unsaturated FAs (FA 14:1, FA 16:1, FA 18:2, FA 18:1). As appraisable from Figure 2, these eleven FAs represent only a very minor part of the total FAs portion present in lanolin. In fact, according to literature, regardless of the degree of unsaturation of the FAs chain, lanolin is composed of many different and atypical FAs (up to 170 species), that span from very long chain FAs (i.e., FAs with over 30 carbon atoms in their chains) to branched FAs, whose standards are not easily acquirable. In the past, some authors engaged in the profiling of acid components of this matrix; it seems that a major part of FAs is composed of iso (22%) and anteiso (26%) fatty acids, though a proper quantitation was never performed [8,9,10,11,12]. Thanks to ^1^H NMR spectroscopy, it is safe to say that portions B and E have a content of almost 100% and 72% of FAs, respectively; therefore, we could speculate that the non-identified species in these two portions (Figure 2A) can be ascribed to the class of FAs, though we cannot suppose whether they are even saturated linear chained FAs. Other than that, it is possible that portions B and E are distinguished by FAs with highly different solubilities. On the other hand, lanolin α-HFAs have a higher chance to be mainly characterized by linear saturated long chains; indeed, the four synthetic standards in our possession (α-hydroxy myristic, palmitic, stearic, and arachidic acids) seem to cover more than half the content of portion D, which, according to ^1^H NMR, does not seem to contain any FAs species. These speculations are all, in fact, supported by ^1^H NMR spectroscopy (Figures S3–S7), which, on one hand, allowed us to qualitatively characterize and semi-quantify the composition of all the different portions and to state that the proposed protocol is capable of selectively separating FAs from α-HFAs and, on the other, did not register any signal related to unsaturated bonds, confirming the negligible presence of unsaturated FAs. The percentages (*w*/*w*) of all identified FAs and α-HFAs in each portion can be found in supporting information Table S1, and exemplificative chromatograms for FAs and α-HFAs are shown in Figures S8 and S9.

## 3. Materials and Methods

### 3.1. Chemicals

Lanolin, FAs standards (lauric (FA 12:0), myristic (FA 14:0), palmitic (FA 16:0), stearic (FA 18:0), arachidic (FA 20:0), behenic (FA 22:0), lignoceric (FA 24:0), myristoleic (FA 14:1), palmitoleic (FA 16:1), linoleic (FA 18:2), and oleic (FA 18:1) acids), internal standard (IS, heptadecanoic acid (FA17:0)), methanol, cyclohexane, potassium hydroxide, hydrochloric acid, N-Trimethylsilyl-N-methyl trifluoroacetamide (MSTFA), trimethylanilinium hydroxide (MethElute™, TMPAH), and deuterated chloroform (CDCl_3_) were acquired from Sigma-Aldrich (Milan, Italy). α-Hydroxy fatty acids (myristic (α-HFA 14:0), palmitic (α-HFA 16:0), and stearic (α-HFA 18:0) were synthetized from the corresponding FAs (FA 14:0, FA 16:0, and FA 18:0) according to recently reported procedures based on the chlorination of FAs with trichloroisocyanuric acid, a versatile green chlorinating agent [19,20], and successive α-hydroxylation [18] or, alternatively, on the enzyme catalysed α-hydroxylation of FAs [21]. The synthetic procedure for α-hydroxy arachidic acid (α-HFA 20:0) can be found in the supporting information (Procedure S1).

### 3.2. Preparation of Standard Solutions and Calibration and Quality Control Samples

Stock standard solutions (25 µg/mL) of FAs, α-HFAs, and IS were stored at −20 °C in the dark. Working solutions were prepared in methanol from stock solutions and used for the preparation of calibration curves and quality control (QC) samples, at the concentrations reported in Table 5.

Calibration and QC levels were prepared daily by adding suitable amounts of working solutions, and the amounts in Table 6 were injected in GC-MS system.

### 3.3. Gas Chromatography Conditions

The determination of FAs and α-HFAs was performed on an Agilent 6890 Plus Gas Chromatograph interfaced with a single quadrupole 5973 N detector (Palo Alto, CA, USA). The GC separation was carried out on an Agilent capillary column CP sil 8 CB (15 m × 0.25 mm i.d., 0.25 µm film thickness). The Mass Spectrometer worked with Electron Ionization (EI) by Single Reaction Monitoring (SIM) mode. The acquisition spectra were elaborated using Agilent ChemStation (Palo Alto, CA, USA). The Quantitative Analysis platform of the Masshunter software (Agilent Technologies) was used to process the analytical data.

#### 3.3.1. Fatty Acids Chromatography

FAs were analysed using the following conditions: from 70 °C to 160 °C at 40 °C/min, and then to 290 °C at 8 °C/min and held at 290 °C for 2 min; injector temperature: 270 °C; ion source temperature: 300 °C; helium was used as carrier gas with a flow set to 1.1 mL/min; injection mode: splitless; injection volume: 2 μL; run time: 22.5 min.

#### 3.3.2. α-Hydroxy Fatty Acids Chromatography

α-HFAs were analysed using the following conditions: 50 °C held for 2 min, then from 50 °C to 300 °C at 20 °C/min, and then held at 300 °C for 3 min; injector temperature: 250 °C; ion source temperature: 300 °C; helium was used as carrier gas with a flow set to 1.0 mL/min; injection mode: splitless; injection volume: 2 μL; run time: 17.5 min.

### 3.4. GC-MS Method Validation

The method was validated according to the guidelines on analytical procedures validation of the European Medicines Agency (EMA) [22]. Linearity, sensitivity and specificity, and precision and accuracy were validated on an Agilent 6890 Plus GC-MS system.

#### 3.4.1. Linearity

Calibration standards were prepared and analysed in duplicate in three independent runs. The calibration curves were constructed by linear regression analysis of the peak area ratios of each compound to the IS against nominal analyte concentration. The correlation was tested over the whole range of concentration to ensure linear regression. Linearity was considered satisfactory if r^2^ ≥ 0.990 and CV ≤ 15%.

#### 3.4.2. Sensitivity and Specificity

The specificity of the assay was evaluated by comparison of GC-MS chromatograms of analytes at the lower limit of quantitation (LLOQ) to those of blank samples in triplicate. The LLOQ was determined as the lowest concentration with values for precision and accuracy within ± 20% and a signal-to-noise (S/N) ratio of the peak areas ≥10, the limit of detection (LOD) as the lowest concentration with a signal-to-noise (S/N) ratio of the peak areas ≥3.

#### 3.4.3. Precision and Accuracy

Precision and accuracy of the method were determined by assaying three replicates of each of the QC samples in three separate analytical runs at low, intermediate, and high level. Precision and accuracy were determined by calculating the percentage coefficient of variation (%CV) and the percentage bias (%BIAS). Precision and accuracy were considered satisfactory if %CV and %BIAS were ≤ 15%.

### 3.5. Lanolin Acid Components Fractioning

Lanolin (10 g) was suspended in 50 mL of an aqueous solution of KOH 10% (*w/v*) and heated at reflux (110 °C) for 48 h. After cooling the mixture, all its unsaponifiable components were extracted with ethyl acetate, and the organic phase was discarded. The alkaline phase was then brought to acidic pH (~1) with HCl 30% and allowed to rest for a few minutes for the precipitation of a portion of the acid components, which was then filtered. The attained solid (A, 3.5 g), was treated with 30 mL of methanol for 2 h: a precipitate was formed and collected (B, 1.2 g) and the methanolic solution was dried under vacuum. The attained waxy solid (C, 2 g) was then treated with 10 mL of cyclohexane overnight, to obtain a precipitate (D, 0.75 g) and a solution, then evaporated under reduced pressure to obtain a solid wax (E, 1 g). See Figure 1 for reference. This procedure was performed three times, in order to have three samples for each portion, for a total of n. 15 samples.

### 3.6. ^1^H NMR Spectroscopy Analysis

^1^H NMR spectra were recorded at 300 MHz using a Varian Mercury 300 FT-NMR spectrometer (Palo Alto, CA, USA). All samples were dissolved in CDCl_3_ at a concentration of approximately 50 mg/mL for ^1^H NMR qualitative analysis. Chemical shifts are reported in ppm relative to residual solvent (CHCl_3_) as internal standard. NMR spectra were processed using the software MestRe Nova (version 14.1, Mestrelab Research S.L., Santiago de Compostela, Spain).

### 3.7. Sample Preparation for Gas Chromatography

#### 3.7.1. Fatty Acids

FAs were dissolved in methanol and diluted to 10 μg/mL. In an autosampler vial, 10 μL of the solution was added with 25 μL of IS (1 μg/mL) and 5 μL of MethElute™ and vortexed, to obtain the correspondent FAMEs.

#### 3.7.2. α-Hydroxy Fatty Acids

α-HFAs were dissolved in methanol and diluted to 10 μg/mL. In an autosampler vial, 30 μL of the solution was added with 15 μL of IS (1 μg/mL) and evaporated under a gentle stream of nitrogen. The residue was reconstituted with 30 μL of MSTFA and heated at 75 °C in oven for 15 min to obtain the correspondent trimethylsilyl derivatives.

## 4. Discussion and Conclusions

To our knowledge, this is one of the first attempts to characterize the constitution in acid components of lanolin. Previous works, in fact, have engaged in the characterization of lanolin as a whole, without focusing on the construction of a profile of acid components [10,11]; only a few authors, more than fifty years ago, were able to provide a panel that included a differentiation of the different acid species, though they did not quantify them [12]. The attained results suggest the presence of a plethora of different FAs involved in the formation of several compounds which are not yet characterized; in fact, the profile of FAs is far from completion, and it is highly speculative to affirm that such a profile may be solely composed of saturated linear-chained FAs. On the other hand, the profile in α-HFAs that we found strongly suggests that sebaceous glands of sheep are more likely to produce saturated and linear α-HFAs. The use of ^1^H NMR spectroscopy was essential to design an efficient separation procedure of the different acid components of lanolin; in fact, even if we were not able to identify all the species in the different portions, we can confidently affirm the effectiveness our protocol, thanks to the detection of the characteristic proton signals ascribable to FAs and α-HFAs. Nevertheless, further studies are necessary to have a comprehensive profile of all species present in this matrix. In this view, we were able to develop a new derivatization method for the conversion of FAs to FAMEs meant for the analysis in GC-MS of FAs, which are receiving more and more attention over the years, particularly for their involvement in the production of biofuels. This method has the chance to be used especially for the characterization of FAs mixtures, allowing a much more rapid derivatization of the analytes in comparison to traditional procedures. Therefore, it could represent an innovation for the analyses of biomasses enriched in FAs and their characterization. Nonetheless, even if not fully characterized, the whole spectrum of lanolin acid components could be a novel source for the production of new materials. In fact, other than their involvement in biofuel manufacturing, FAs are important substrates used in the cosmetic and polymers industries [23].

## Figures and Tables

**Table 1:** Calibration ranges for selected analytes.

Compound	ng Injected (ng inj)
FA 12:0	0.125–5
FA 14:0	0.125–5
FA 16:0	0.5–10
FA 18:0	0.5–10
FA 20:0	0.125–5
FA 22:0	0.125–5
FA 24:0	0.5–10
FA 14:1	0.5–10
FA 16:1	0.5–10
FA 18:2	0.5–10
FA 18:1	0.5–10
α-HFA 14:0	0.5–20
α-HFA 16:0	0.5–20
α-HFA 18:0	0.5–20
α-HFA 20:0	0.5–20

**Table 2:** Calibration parameters for selected analytes.

Compound	r^2^	CV (%)	LOD (ng inj)	LOQ (ng inj)
FA 12:0	0.999	10.2	0.02	0.08
FA 14:0	0.998	11.1	0.02	0.05
FA 16:0	0.997	14.0	0.01	0.04
FA 18:0	0.999	13.5	0.09	0.28
FA 20:0	0.999	8.5	0.05	0.18
FA 22:0	0.999	14.4	0.03	0.09
FA 24:0	0.999	13.8	0.08	0.26
FA 14:1	0.994	14.6	0.10	0.33
FA 16:1	0.999	5.1	0.11	0.36
FA 18:2	0.998	9.9	0.08	0.28
FA 18:1	0.998	14.2	0.10	0.32
α-HFA 14:0	0.996	12.0	0.04	0.13
α-HFA 16:0	0.999	10.4	0.05	0.16
α-HFA 18:0	0.997	12.1	0.10	0.34
α-HFA 20:0	0.997	11.2	0.09	0.30

**Table 3:** Precision (%CV) and accuracy (%BIAS) for selected analytes.

Compound	Amount (ng inj)	Precision (%)	Accuracy (%)
FA 12:0	0.125	5.0	6.8
	0.5	2.5	6.8
	5	3.7	2.3
FA 14:0	0.125	3.1	11.9
	0.5	4.1	3.5
	5	2.9	0.7
FA 16:0	0.5	4.9	14.7
	2.5	11.8	12.1
	10	4.1	2.8
FA 18:0	0.5	3.9	4.3
	2.5	4.1	5.7
	10	7.1	1.3
FA 20:0	0.125	8.6	8.2
	0.5	1.8	11.8
	5	4.7	2.5
FA 22:0	0.125	5.5	3.7
	0.5	10.3	9.7
	5	13.9	2.9
FA 24:0	0.5	8.3	10.3
	2.5	7.2	4.7
	10	1.4	1.0
FA 14:1	0.5	10.9	14.0
	2.5	10.5	10.1
	10	3.5	2.4
FA 16:1	0.5	7.1	8.1
	2.5	14.1	12.4
	10	4.8	8.1
FA 18:2	0.5	2.2	10.6
	2.5	7.0	4.9
	10	3.9	12.4
FA 18:1	0.5	4.1	5.4
	2.5	7.1	6.4
	10	2.8	5.3
α-HFA 14:0	0.5	2.4	11.3
	5	4.3	9.4
	20	14.8	0.5
α-HFA 16:0	0.5	3.7	14.7
	5	14.4	12.3
	20	11.2	1.6
α-HFA 18:0	0.5	8.5	3.8
	5	1.6	11.3
	20	3.2	3.1
α-HFA 20:0	0.5	14.8	5.3
	5	4.6	7.3
	20	3.7	1.8

**Table 4:** Summary of the features of portions A–E. The molar fraction between α-HFAs and FAs was evaluated by ^1^H NMR spectroscopy analysis and calculated taking into account the integral values of the characteristic peaks of FAs and α-HFAs.

		Molar Fraction	
Portion	Weight (g)	α-HFAs	FAs
A	3.5	0.43	0.57
B	1.2	0	1
C	2	0.52	0.48
D	0.75	1	0
E	1	0.28	0.72

**Table 5:** Concentrations of the different stock solutions.

Stock Solution	Concentration (µg/mL)
FAs	10
α-HFAs	10
IS	1

**Table 6:** Amounts of analytes injected in the GC-MS system for QC. An “×” symbol was placed in the cells corresponding to the different amounts that were injected for each analyte.

	Injected Amount (ng)									
Compound	0.125	0.25	0.5	1.0	1.25	2	2.5	5	10	20
FA 12:0	×	×	×	×			×	×		
FA 14:0	×	×	×	×			×	×		
FA 16:0			×	×			×	×	×	
FA 17:0 (IS)					×					
FA 18:0			×	×			×	×	×	
FA 20:0	×	×	×	×			×	×		
FA 22:0	×	×	×	×			×	×		
FA 24:0			×	×			×	×	×	
FA 14:1			×	×			×	×	×	
FA 16:1			×	×			×	×	×	
FA 18:2			×	×			×	×	×	
FA 18:1			×	×			×	×	×	
α-HFA 14:0			×	×		×		×	×	×
α-HFA 16:0			×	×		×		×	×	×
α-HFA 18:0			×	×		×		×	×	×
α-HFA 20:0			×	×		×		×	×	×

## Data Availability

Not applicable.

## Supplementary Materials

The following supporting information can be downloaded at: https://www.mdpi.com/article/10.3390/molecules28041635/s1, Procedure S1: Synthesis of α-hydroxy arachidic acid; Figure S1: ^1^H NMR spectrum of FAs in CDCl_3_. The characteristic peak of FAs is circled in red; Figure S2: ^1^H NMR spectrum of α-HFAs in CDCl_3_. The characteristic peak of α-HFAs is circled in blue; Figure S3: ^1^H NMR spectrum of fractioning portion A in CDCl_3_; Figure S4: ^1^H NMR spectrum of fractioning portion B in CDCl_3_; Figure S5: ^1^H NMR spectrum of fractioning portion C in CDCl_3_; Figure S6: ^1^H NMR spectrum of fractioning portion D in CDCl3; Figure S7: ^1^H NMR spectrum of fractioning portion E in CDCl3; Figure S8 GC-MS SIM chromatogram of portion B, exemplificative for FAs analysis; Figure S9 GC-MS SIM chromatogram of portion D, exemplificative for α-HFAs analysis. Table S1: Mean percentage (*w*/*w*) composition of the analytes in each portion of lanolin. A–E refer to the different portions of lanolin, whereas A is composed of a mixture of FAs and α-HFAs, B of almost exclusively FAs, C of a mixture of FAs and α-HFAs, D of solely α-HFAs, and E of mainly FAs. See Figure 1 (main text) for reference.

## References

[1] Hamish F., Tom Q., Clementine O. (2021). Food Waste Index Report 2021.

[2] Ramos J.L., García-Lorente F., Valdivia M., Duque E. (2017). Green Biofuels and Bioproducts: Bases for Sustainability Analysis. Microb. Biotechnol..

[3] Sánchez Muñoz S., Rocha Balbino T., Mier Alba E., Gonçalves Barbosa F., Tonet de Pier F., Lazuroz Moura de Almeida A., Helena Balan Zilla A., Antonio Fernandes Antunes F., Terán Hilares R., Balagurusamy N. (2022). Surfactants in Biorefineries: Role, Challenges & Perspectives. Bioresour. Technol..

[4] Campos de Bomfim A.S., Oliveira D.M.d., Voorwald H.J.C., Benini K.C.C.d.C., Dumont M.J., Rodrigue D. (2022). Valorization of Spent Coffee Grounds as Precursors for Biopolymers and Composite Production. Polymers.

[5] Sawangkeaw R., Ngamprasertsith S. (2013). A Review of Lipid-Based Biomasses as Feedstocks for Biofuels Production. Renew. Sustain. Energy Rev..

[6] Petek B., Marinšek Logar R. (2021). Management of Waste Sheep Wool as Valuable Organic Substrate in European Union Countries. J. Mater. Cycles Waste Manag..

[7] Reutelingsperger C.M.H.G., Erutan B.V. (2013). A Method to Wash Greasy Wool, a Method to Separate Lanolin from the Said Greasy Wool, Wool and Lanolin Obtainable by These Methods. U.S. Patent.

[8] Domínguez C., Jover E., Garde F., Bayona J.M., Erra P. (2003). Characterization of Supercritical Fluid Extracts from Raw Wool by TLC-FID and GC-MS. J. Am. Oil Chem. Soc..

[9] Scanes C.G. (2018). Animal Attributes Exploited by Humans (Nonfood Uses of Animals). Animals and Human Society.

[10] Rissmann R., Oudshoorn M.H.M., Kocks E., Hennink W.E., Ponec M., Bouwstra J.A. (2008). Lanolin-Derived Lipid Mixtures Mimic Closely the Lipid Composition and Organization of Vernix Caseosa Lipids. Biochim. Biophys. Acta–Biomembr..

[11] Jover E., Adahchour M., Bayona J.M., Vreuls R.J.J., Brinkman U.A.T. (2005). Characterization of Lipids in Complex Samples Using Comprehensive Two-Dimensional Gas Chromatography with Time-of-Flight Mass Spectrometry. J. Chromatogr. A.

[12] Sengupta A., Behera J. (2014). Comprehensive View on Chemistry, Manufacturing & Applications of Lanolin Extracted from Wool Pretreatment. Am. J. Eng. Res..

[13] Dei Cas M., Paroni R., Saccardo A., Casagni E., Arnoldi S., Gambaro V., Saresella M., Mario C., La Rosa F., Marventano I. (2020). A Straightforward LC-MS/MS Analysis to Study Serum Profile of Short and Medium Chain Fatty Acids. J. Chromatogr. B Anal. Technol. Biomed. Life Sci..

[14] Wu Z., Zhang Q., Li N., Pu Y., Wang B., Zhang T. (2017). Comparison of Critical Methods Developed for Fatty Acid Analysis: A Review. J. Sep. Sci..

[15] Chiu H.H., Kuo C.H. (2020). Gas Chromatography-Mass Spectrometry-Based Analytical Strategies for Fatty Acid Analysis in Biological Samples. J. Food Drug Anal..

[16] Kramer J.K.G., Fellner V., Dugan M.E.R., Sauer F.D., Mossoba M.M., Yurawecz M.P. (1997). Evaluating Acid and Base Catalysts in the Methylation of Milk and Rumen Fatty Acids with Special Emphasis on Conjugated Dienes and Total Trans Fatty Acids. Lipids.

[17] Murrieta C.M., Hess B.W., Rule D.C. (2003). Comparison of Acidic and Alkaline Catalysts for Preparation of Fatty Acid Methyl Esters from Ovine Muscle with Emphasis on Conjugated Linoleic Acid. Meat Sci..

[18] Bertolini V., Pallavicini M., Tibhe G., Roda G., Arnoldi S., Monguzzi L., Zoccola M., Di Nardo G., Gilardi G., Bolchi C. (2021). Synthesis of α-Hydroxy Fatty Acids from Fatty Acids by Intermediate α-Chlorination with TCCA under Solvent-Free Conditions: A Way to Valorization of Waste Fat Biomasses. ACS Omega.

[19] Bolchi C., Valoti E., Straniero V., Ruggeri P., Pallavicini M. (2014). From 2-Aminomethyl-1,4-Benzodioxane Enantiomers to Unichiral 2-Cyano- and 2-Carbonyl-Substituted Benzodioxanes via Dichloroamine. J. Org. Chem..

[20] Pallavicini M., Bolchi C., Fumagalli L., Piccolo O., Valoti E. (2011). Highly Efficient Racemisation of a Key Intermediate of the Antibiotic Moxifloxacin. Tetrahedron Asymmetry.

[21] Giuriato D., Correddu D., Catucci G., Di Nardo G., Bolchi C., Pallavicini M., Gilardi G. (2022). Design of a H_2_O_2_-Generating P450SPα Fusion Protein for High Yield Fatty Acid Conversion. Protein Sci..

[22] European Medicines Agency ICH Q2(R2) Validation of Analytical Procedures—Scientific Guideline. https://www.ema.europa.eu/en/ich-q2r2-validation-analytical-procedures-scientific-guideline.

[23] Biermann U., Bornscheuer U.T., Feussner I., Meier M.A.R., Metzger J.O. (2021). Fatty Acids and Their Derivatives as Renewable Platform Molecules for the Chemical Industry. Angew. Chem. Int. Ed..

