# Supplementary material for: Fractioning and Compared 1H NMR and GC-MS Analyses of Lanolin Acid Components
Source: Molecules. 2023 Feb 8;28(4):1635. doi: 10.3390/molecules28041635 (PMC9964252; doi:10.3390/molecules28041635)
Supplement: Supplementary file 1 [file molecules-28-01635-s001.zip › molecules-2161019-supplementary.pdf]

Fractioning and compared ^{1}H NMR and GC-MS analyses of lanolin acid components

Camillo Morano [1], Michele Dei Cas [2], Roberta Bergamaschi [3], Erika Palmisano [3], Marco Pallavicini [1], Cristiano Bolchi [1], Gabriella Roda [1] and Sara Casati [3,*]

[1] Department of Pharmaceutical Sciences, Università degli Studi di Milano, Milan, Italy

[2] Department of Health Sciences, Università degli Studi di Milano, Milan, Italy

[3] Department of Biomedical, Surgical and Dental Sciences, Università degli Studi di Milano, Milan, Italy

* Correspondence: sara.casati@unimi.it

Supporting Information

Procedure S1 Synthesis of α-hydroxy arachidic acid.

Figure S1 ^{1}H NMR spectrum of FAs in $CDCl_3$. The characteristic peak of FAs is circled in red.

Figure S2 ^{1}H NMR spectrum of α-HFAs in $CDCl_3$. The characteristic peak of α-HFAs is circled in blue.

Figure S3 ^{1}H NMR spectrum of fractioning portion A in $CDCl_3$.

Figure S4 ^{1}H NMR spectrum of fractioning portion B in $CDCl_3$.

Figure S5 ^{1}H NMR spectrum of fractioning portion C in $CDCl_3$.

Figure S6 ^{1}H NMR spectrum of fractioning portion D in $CDCl_3$.

Figure S7 ^{1}H NMR spectrum of fractioning portion E in $CDCl_3$.

Figure S8 GC-MS SIM chromatogram of portion B, exemplificative for FAs analysis.

Figure S9 GC-MS SIM chromatogram of portion D, exemplificative for α-HFAs analysis.**Table S1** Mean percentage (w/w) composition of the analytes in each portion of lanolin. A-E refer to the different portions of lanolin, whereas A is composed of a mixture of FAs and α-HFAs, B of almost exclusively FAs, C of a mixture of FAs and α-HFAs, D of solely α-HFAs and E of mainly FAs. See Figure 1 (main text) for reference.

Procedures

Procedure S1 Synthesis of α-hydroxy arachidic acid.

In a round-bottom flask wrapped in aluminium foil, arachidic acid (35 mmol) was heated under magnetic stirring at 80 °C until melting. The liquid mass was added of PCl3 (1.2 mmol, 104 μL) and left reacting for 1 h. TCCA (16.3 mmol, 3.78 g) was slowly added over 30 min. The mixture was then stirred for 24 h at 80 °C. After this time, the reaction mixture was cooled down at room temperature, and ethyl acetate was added: a white precipitate was formed. The solid was then removed by filtration, and the filtrate was washed with a solution of sodium metabisulfite (10% w/v) and with brine. The organic layer was collected, dried over sodium sulfate, and concentrated under vacuum to give the desired alpha-chloro acid. In a round-bottom flask, KOH (140 mmol, 7.85 g) and water (200 mL) were stirred at 80 °C for 30 min. The crude 2-chloro arachidic acid resultant from the previous step was added to the KOH water solution, and the mixture was refluxed for 24 h. Then, the mixture was cooled down at room temperature and the pH was adjusted to 1 using HCl 1 M. A white solid precipitated. The mixture was filtered, and the solid was recovered. After purification by trituration with acetonitrile, in a 1:3 ratio, α-hydroxy arachidic acid was obtained as a white solid with a final yield of 67%. mp 89.2 °C, Rf (dichloromethane/methanol 9:1) = 0.65, 1H NMR (300 MHz, $CDCl_3$): δ 4.28 (dd, J = 7.4, 4.2 Hz, 1H), 1.90−1.63 (m, 2H), 1.49–1.18 (m, 32H), 0.87 (t, J = 6.8 Hz, 3H). ESI negative MS: calcd for $C_{20}H_{40}O_3$ $[M-H]^-$, m/z, 327.2904; found, 327.3.

Figures

Figure S1 ^{1}H NMR spectrum of FAs in $CDCl_3$. The characteristic peak of FAs is circled in red.

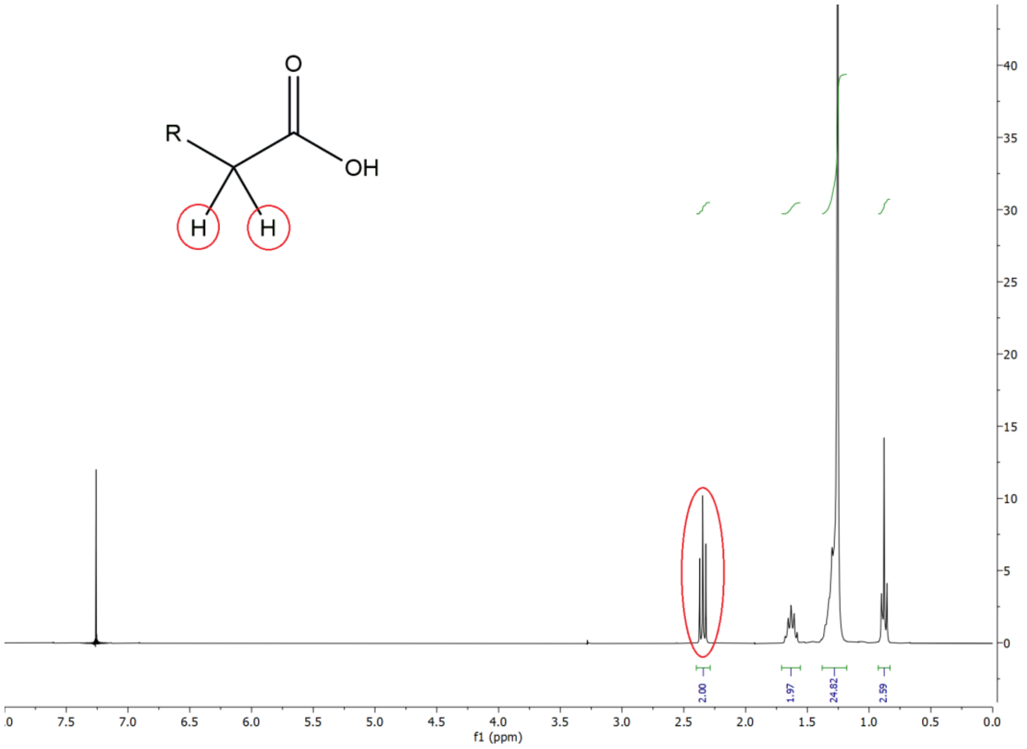

Figure S2 ^{1}H NMR spectrum of α-HFAs in $CDCl_3$. The characteristic peak of α-HFAs is circled in blue.

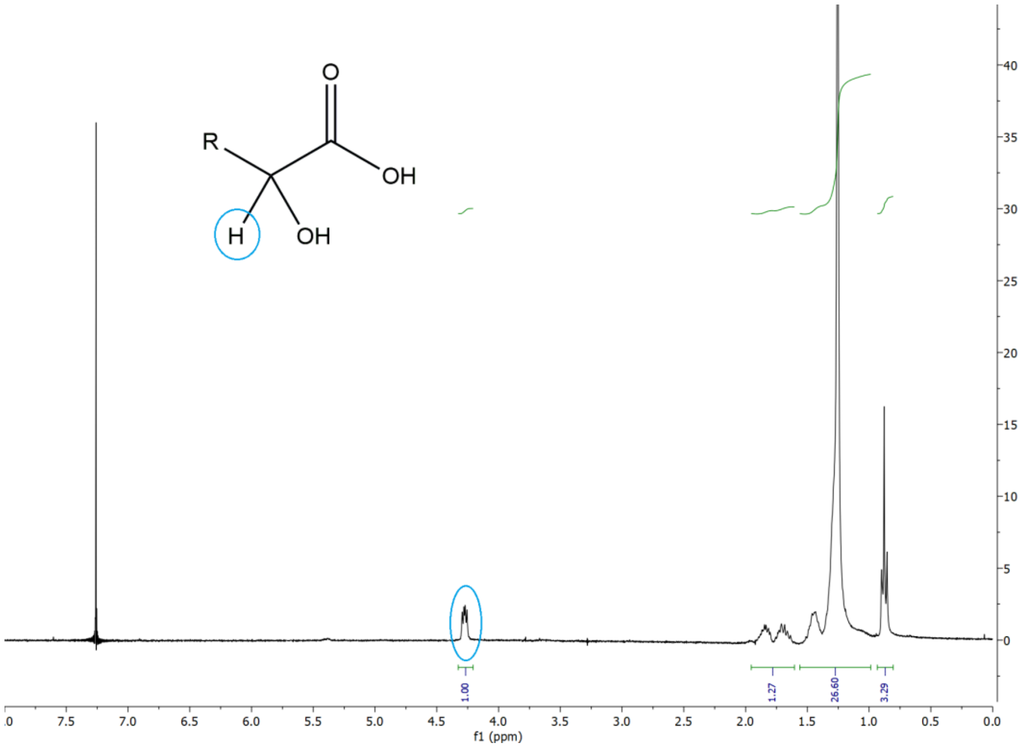

Figure S3 ^{1}H NMR spectrum of fractioning portion A in $CDCl_3$.

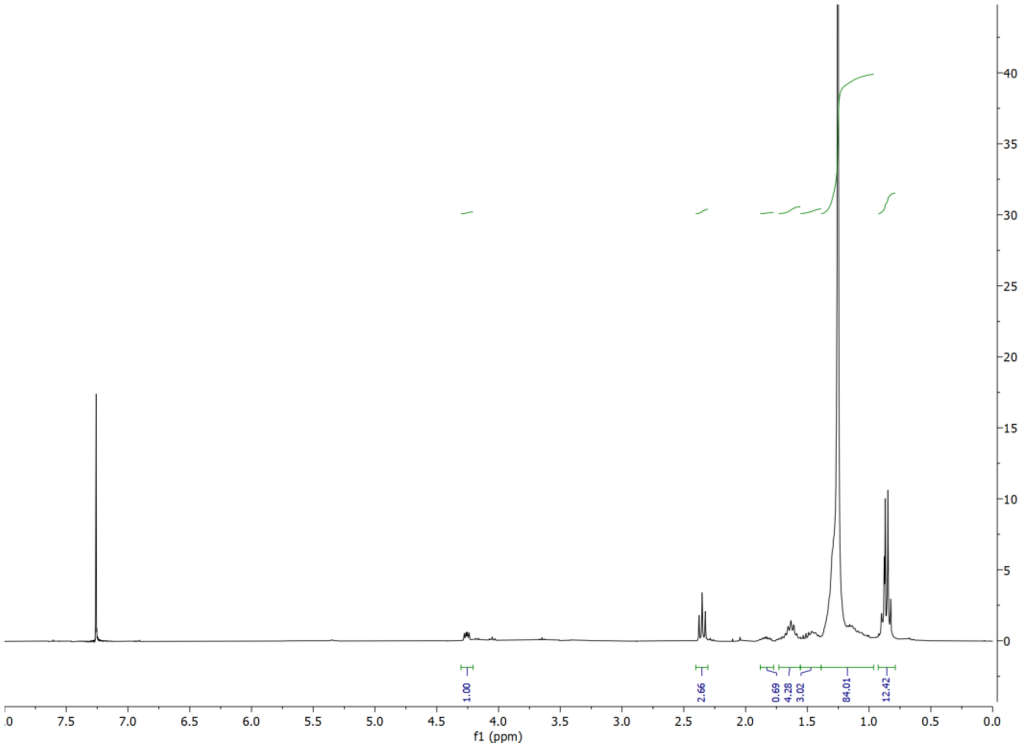

Figure S4 ^{1}H NMR spectrum of fractioning portion B in $CDCl_3$.

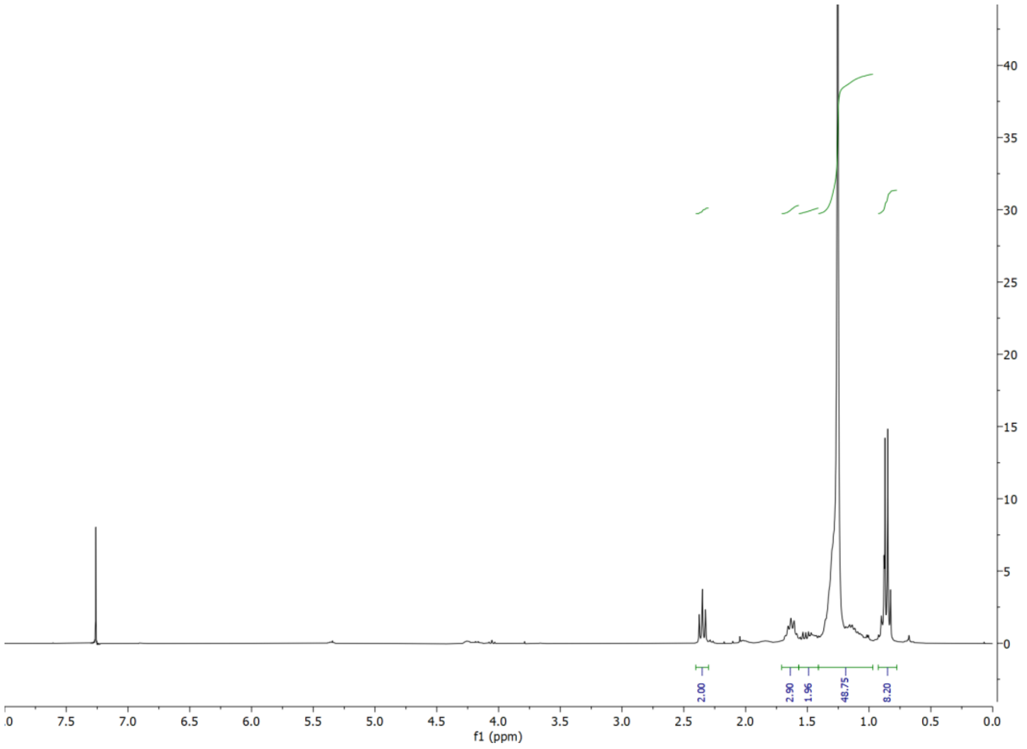

Figure S5 ^{1}H NMR spectrum of fractioning portion C in $CDCl_3$.

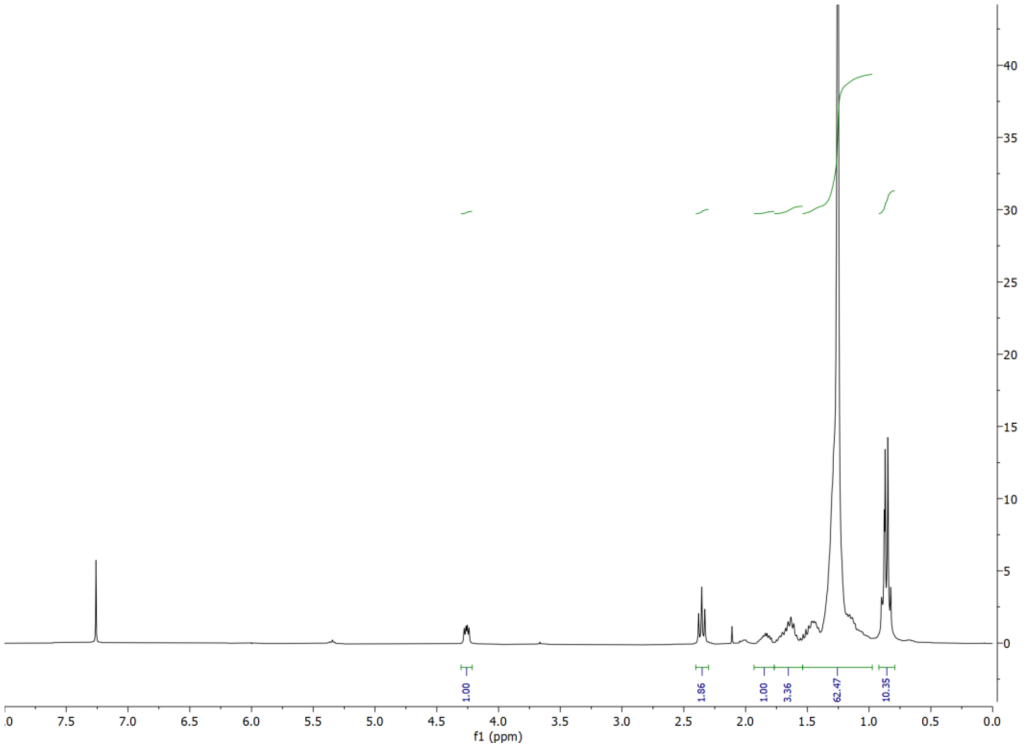

Figure S6 ^{1}H NMR spectrum of fractioning portion D in $CDCl_3$.

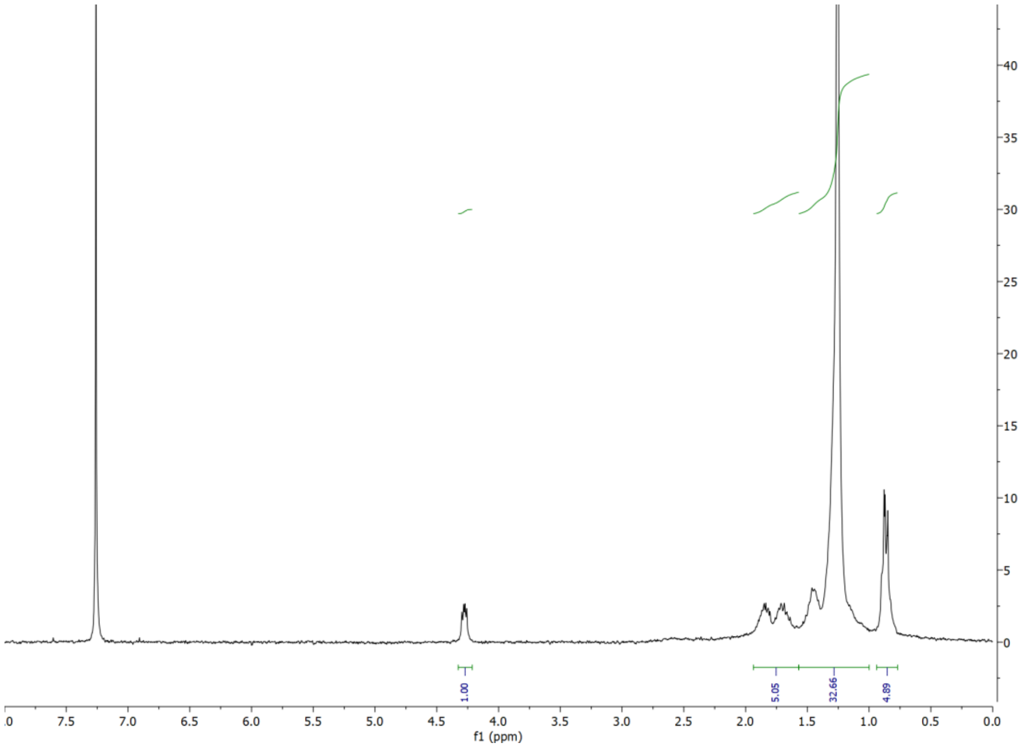

Figure S7 ^{1}H NMR spectrum of fractioning portion E in $CDCl_3$.

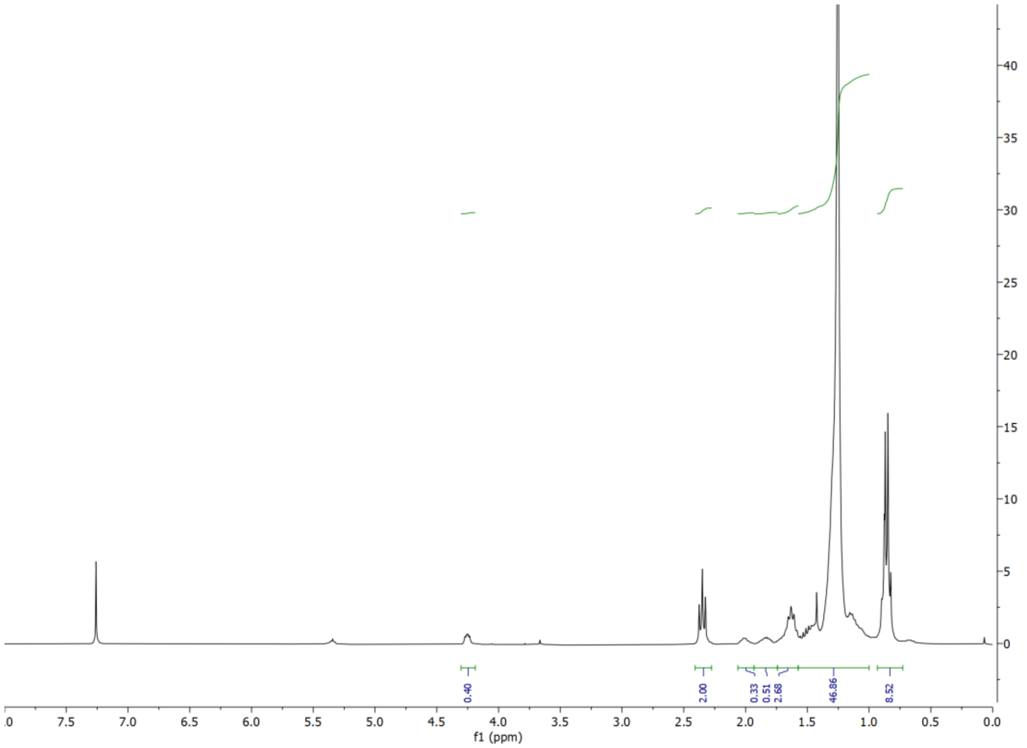

Figure S8 GC-MS SIM chromatogram of portion B, exemplificative for FAs analysis.

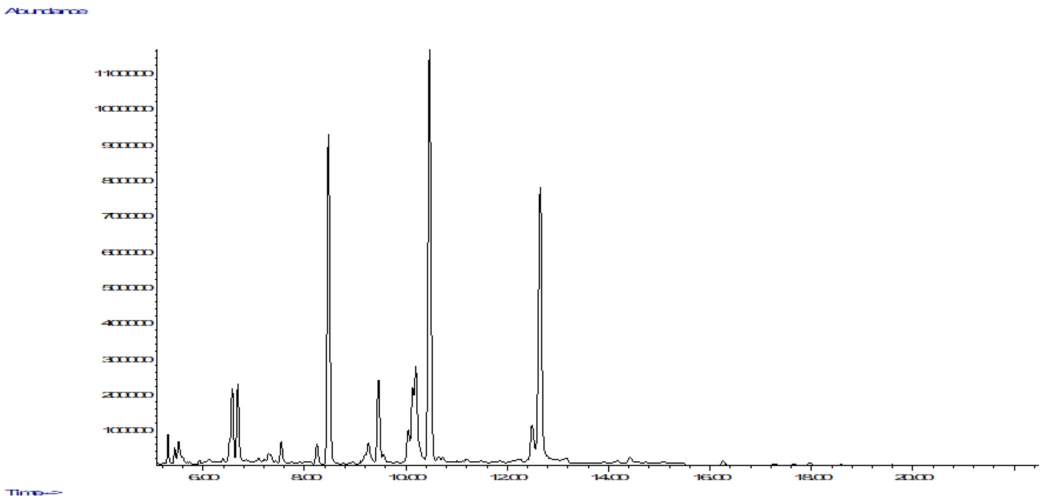

Figure S9 GC-MS SIM chromatogram of portion D, exemplificative for α-HFAs analysis.

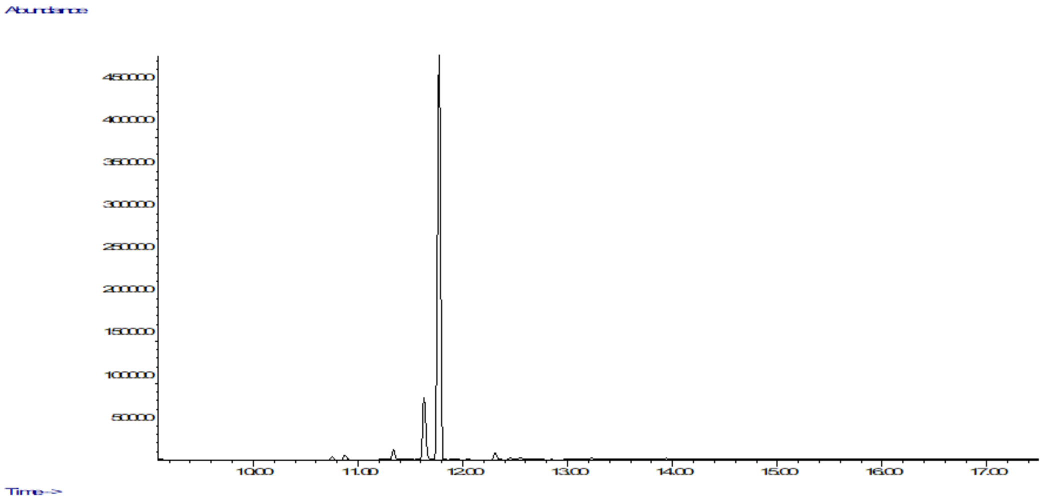

Tables

Table S1 Mean percentage (w/w) composition of the analytes in each portion of lanolin. A-E refer to the different portions of lanolin, whereas A is composed of a mixture of FAs and α-HFAs, B of almost exclusively FAs, C of a mixture of FAs and α-HFAs, D of solely α-HFAs and E of mainly FAs. See Figure 1 (main text) for reference.

	**A**	**B**	**C**	**D**	**E**
**FA 12:0**	0.90	0.00	1.07	0.93	1.03
**FA 14:0**	1.00	0.00	0.13	0.00	0.23
**FA 16:0**	4.30	0.00	0.70	0.00	0.40
**FA 18:0**	7.00	3.37	1.17	1.47	3.23
**FA 20:0**	0.57	0.63	0.53	0.43	0.80
**FA 22:0**	1.33	1.33	1.23	1.20	1.37
**FA 24:0**	2.00	2.23	0.80	0.53	1.30
**FA 14:1**	1.50	1.43	0.90	1.17	0.97
**FA 16:1**	1.57	0.57	1.07	0.63	0.77
**FA 18:2**	0.00	0.00	0.00	0.00	0.00
**FA 18:1**	3.65	0.73	0.47	0.40	0.83
**α-HFA 14:0**	1.12	0.97	2.37	3.20	1.60
**α-HFA 16:0**	8.40	1.03	20.27	42.97	8.55
**α-HFA 18:0**	1.15	0.80	3.18	3.47	1.30
**α-HFA 20:0**	8.28	6.13	11.22	7.88	6.90
**Total FAs**	23.81	10.30	8.07	6.77	10.93
**Total α-HFAs**	18.95	8.93	37.03	57.52	18.35
